# A New Gilded Age, and What It Means for Global Health: Comment on "Global Health Governance Challenges 2016 – Are We Ready?"

**DOI:** 10.15171/ijhpm.2016.115

**Published:** 2016-08-17

**Authors:** Ted Schrecker

**Affiliations:** School of Medicine, Pharmacy and Health, Durham University, Durham, UK.

**Keywords:** Global Health Politics, Political Economy, Neoliberalism, Inequality

## Abstract

New contours of global inequality present new challenges for global health, and require that we consider new kinds of health issues as global. I provide a number of illustrations, arguing the need for a political science of health that goes beyond conventional preoccupations with formal institutional and inter-state interactions and takes into account how globalization has affected the health policy landscape and restructured the distribution of economic and political power not only among countries, but also within them.

As Prof. Kickbusch points out,^1^ with respect to health in foreign policy the recent past has several encouraging features. Donor assistance for health quadrupled in value over a quarter-century, representing a major achievement on the part of the international community, and its value has not collapsed in the wake of the financial crisis as some observers had feared.^2^ Failures of coordinated action to prevent the spread of antimicrobial resistance, arguably the clearest example of the need for collective action to supply a global public good for health, have not yet led to health catastrophe although the peril remains real.^3^ However, the landscape of health foreign policy, especially when considered principally in terms of the visible actions of national governments and other high-profile actors – at international conferences, through multilateral agreements and similar formal routines – resembles a pretty seafront that hides treacherous undercurrents and invisible toxic hazards. Prof. Kickbusch laudably directs our attention to the “new landscape of inequality” and recognizes that “the challenge ahead is…deeply political.”^1^ Indeed, it is. This commentary concentrates on such political challenges, with the aim of adding a dimension to Prof. Kickbusch’s analysis.

Today’s and tomorrow’s health policy and politics cannot be understood without reference to the global reorganization of production that has taken place over the past few decades, driven by transnational corporations and allied rich country governments.^4,5^ Health foreign policy analysis and practice have seldom engaged adequately with (for example) the potentially constraining effects of ‘mega-regional’ trade agreements and bilateral investment treaties that give transnational corporate investors a separate, parallel channel of influence on public policy through investor-state dispute settlement (ISDS) provisions.^6^ Discussions of trade policy impacts on health often presume that conflicting priorities can be addressed by working together to achieve “policy coherence”^7^; this Panglossian perspective neglects the possibility that expanding opportunities for profit through global economic integration and reducing health inequities are mutually exclusive, or at the very least cannot be reconciled within existing institutional contexts or distributions of political resources. And the debilitating effects of capital flight and tax avoidance on resources available for the widely accepted objective of universal health coverage (where national governments are genuinely committed to this objective), and more generally on social protection and broader structural issues like inequality,^8,9^ are normally ignored in discussions of how to improve global governance for health.

Health in foreign policy is only part of the global health domain; an additional element involves the interplay of global and domestic (within-border) interests and policy commitments. Partly as a consequence of globalization and associated shifts in political priorities and allegiances, socio-economically patterned divides in health outcomes and access to the prerequisites for living a healthy life now are as deep within many countries as among them – a phenomenon that can usefully be described in terms of “neoliberal epidemics.”^10^ In the North of England municipality where I live and work, battered first by policy-driven deindustrialization and then by social policy retrenchment, differences in male life expectancy at birth between the least and most deprived small areas are comparable to national average differences between the United Kingdom and Senegal.^11^ Even before the financial crisis and subsequent ratcheting-up of inequality in the United States, the Eight Americas study by Murray and colleagues found that “tens of millions of Americans are experiencing levels of health that are more typical of middle-income or low-income developing countries.”^12^ In such countries, “lagoons of wealth and privilege” are often “surrounded by oceans of poverty and mass misery, often divided only, and literally, by the very best security systems that social control technology can buy.”^13^ This description, familiar from an extensive urban studies literature, clearly fits some of the BRICS (Brazil, Russia, India, China, and South Africa) and MINT (Mexico, Indonesia, Nigeria, and Turkey) countries whose domestic policies Prof. Kickbusch correctly identifies as pivotal for prospects of “global health convergence,” and for that matter portions of the high-income world, thereby suggesting new categories of ‘global health’ issues.

We are now in a new Gilded Age that is reminiscent of the end of the nineteenth century in how wealth and deprivation are distributed and concentrated within national borders.^14-16^ Global distributions are also changing; for example, there are now more “ultra high net worth individuals” in China than in the United Kingdom or Germany, although the United States still tops this league table.^17^ Against this background, assumptions about future health trajectories must be rethought, literal and metaphorical maps redrawn with reference to what Robinson^13^ describes as “social” rather than “territorial cartographies” that describe how unequal distributions of power and resources within political boundaries are connected to changing global organizations of production and finance and associated shifts in political influence. In parallel, a newly critical perspective on states and their commitment (or lack of commitment) to the health and well-being of their populations is needed. Some governments use policy instruments such as progressive taxation to finance extension of health services,^18^ calling into question the claim that the imperatives of economic competitiveness preclude such strategies. Other governments appear willing to regard the health of some proportion of their populations as collateral damage from the quest for globalization-related prosperity.^19^ How can we predict which direction a particular state will follow, and improve the effectiveness of state actors with a genuine commitment to reducing health inequalities?

A further concern involves invocations of “the health of the planet” (in Prof. Kickbusch’s words, and those of many others) that fail directly to address distributional politics – eloquently described by Enzensberger^21^ in terms of “that little difference between first class and steerage, between the bridge and the engine room” of Spaceship Earth. The difference is growing. Who will bear the costs of adjustment to environmental constraints? Rarely if ever have the privileged done so. In the case of climate change, the most familiar (although not the only) relevant global environmental challenge: can routine reliance on fossil-fuelled auto transport be cast worldwide as antisocial and health-destructive in the same way as smoking in public places?^22^ Can necessary low-carbon infrastructure investments to retreat from a fossil fuel-centred world be mobilized, and binding carbon pricing regimes be implemented? Can the US$5 trillion in cash now in the hands of transnational corporations^23^ be mobilized in support of the Sustainable Development Goals (SDGs) in ways that do not simply involve socializing the costs of accumulating private profit?

These are core questions for the future of global health, broadly defined. Too little research illuminates the conditions under which health and well-being even within a country’s borders, much less half a world away, will be subordinated to the priorities of elites or political coalitions “concerned to maintain a specific distribution of resources that subordinates labour and preserves elite privileges.”^20^ The need for a political science of health has been widely noted,^24-27^ without substantial take-up in the relevant research communities, although recent work by Kelsall and colleagues using the concept of political settlements^28^ and (outside the health policy field) by Teichman on the politics of social protection^29,30^ offers important methodological advances. Prof. Kickbusch does the valuable service of showing that in the global frame of reference, the need for a political science of health is more urgent than ever.

## Ethical issues

Not applicable.

## Competing interests

Author declares that he has no competing interests.

## Author’s contribution

TS is the single author of the paper.
